# Tranilast-induced stress alleviation in solid tumors improves the efficacy of chemo- and nanotherapeutics in a size-independent manner

**DOI:** 10.1038/srep46140

**Published:** 2017-04-10

**Authors:** Panagiotis Papageorgis, Christiana Polydorou, Fotios Mpekris, Chrysovalantis Voutouri, Eliana Agathokleous, Constantina P. Kapnissi-Christodoulou, Triantafyllos Stylianopoulos

**Affiliations:** 1 Cancer Biophysics Laboratory, Department of Mechanical and Manufacturing Engineering, University of Cyprus, Nicosia, Cyprus; 2 Department of Life Sciences, Program in Biological Sciences, European University Cyprus, Nicosia, Cyprus; 3 Department of Chemistry, University of Cyprus, Nicosia, Cyprus

## Abstract

Accumulation of mechanical stresses during cancer progression can induce blood and lymphatic vessel compression, creating hypo-perfusion, hypoxia and interstitial hypertension which decrease the efficacy of chemo- and nanotherapies. Stress alleviation treatment has been recently proposed to reduce mechanical stresses in order to decompress tumor vessels and improve perfusion and chemotherapy. However, it remains unclear if it improves the efficacy of nanomedicines, which present numerous advantages over traditional chemotherapeutic drugs. Furthermore, we need to identify safe and well-tolerated pharmaceutical agents that reduce stress levels and may be added to cancer patients’ treatment regimen. Here, we show mathematically and with a series of *in vivo* experiments that stress alleviation improves the delivery of drugs in a size-independent manner. Importantly, we propose the repurposing of tranilast, a clinically approved anti-fibrotic drug as stress-alleviating agent. Using two orthotopic mammary tumor models, we demonstrate that tranilast reduces mechanical stresses, decreases interstitial fluid pressure (IFP), improves tumor perfusion and significantly enhances the efficacy of different-sized drugs, doxorubicin, Abraxane and Doxil, by suppressing TGFβ signaling and expression of extracellular matrix components. Our findings strongly suggest that repurposing tranilast could be directly used as a promising strategy to enhance, not only chemotherapy, but also the efficacy of cancer nanomedicine.

The rationale for the use of macromolecules and nanomedicines to treat cancer is based on the hyper-permeability of some tumor blood vessels that allows large therapeutic agents to selectively accumulate into the tumor tissue, and the dysfunction of intratumoral lymphatic vessels that retain the drugs into the tumor for a longer time^1^,^2^,^3^. This phenomenon, known as the Enhanced Permeability and Retention (EPR) effect, forms the basis for the passive delivery of drugs to solid tumors^4^,^5^. Enhanced permeability of tumor blood vessels, however, might cause excessive fluid loss from the vascular to the interstitial space of the tumor reducing blood vessel perfusion and raising the interstitial fluid pressure (IFP)^6^. Hypo-perfusion can drastically decrease the amount of systemically administered drugs that reach the tumor, while interstitial hypertension eliminates pressure gradients across the tumor vessel wall rendering diffusion the dominant mechanism of transvascular transport of drugs^7^,^8^. Furthermore, diffusion is a size-dependent transport mechanism, inversely related to the size of the drug^9^,^10^. Therefore, passive delivery of drugs to solid tumors utilizing the EPR effect is size-dependent and drugs larger than 50 nm in diameter might exhibit poor accumulation and penetration into the tumor^11^,^12^,^13^,^14^.

In previous research, it has been shown that normalization of tumor blood vessels with judicial use of anti-angiogenic agents improves the delivery and thus, the efficacy of macromolecules and small nanomedicines, up to 12 nm in size^10^,^15^,^16^. Recently, it was found that this strategy might improve the efficacy of particles as large as 40 nm^17^. Inability of anti-angiogenic therapies to improve drug efficacy in a size-independent manner is owing to the fact that vascular normalization repairs the abnormally large pores in the tumor blood vessel wall making them less permeable. Decreasing vessel wall pore size reduces IFP but at the same time prevents large nanoparticles from crossing the tumor vessel wall into the tumor interior. Furthermore, vascular normalization treatment cannot treat compressed tumor blood vessels, which are the result of the accumulation of mechanical stresses in the tumor owing to its growth in the confined space of the host tissue^18^,^19^,^20^. Blood vessel compression is another factor that drastically affects tumor perfusion as it reduces the capacity of a vessel to carry blood. This explains why anti-angiogenic agents have failed in desmoplastic tumors, such as pancreatic and breast cancers, which exhibit high stress levels leading to abundant compressed vessels^21^,^22^.

Recently, we found that a major contributor to the accumulation of mechanical stresses in tumors are extracellular matrix (ECM) components, namely collagen and hyaluronan^23^,^24^,^25^. Depletion of these components has been mainly considered to improve the interstitial distribution of nanoparticles, but whether it can improve the efficacy of systemically administered drugs in a size-independent manner remains unexplored^26^,^27^,^28^. Additionally, even though it has been shown that depletion of collagen and hyaluronan using angiotensin inhibitors can improve the efficacy of chemotherapeutics by alleviation of mechanical stresses and decompression of tumor blood vessels^29^, this principle is still not well defined and there is yet no simulation-based or experimental evidence to demonstrate the effect of this approach using drugs of different sizes. Finally, angiotensin inhibitors are commonly used as anti-hypertensive drugs and thus, might affect the blood pressure of patients. Therefore, we urgently need to identify other safe and well tolerated pharmaceutical agents that reduce stress levels and may be added to cancer patients’ treatment regimen^30^,^31^.

To this end, using mathematical modeling and *in vivo* experiments we show that stress alleviation improves the delivery of drugs in a size-independent manner. Furthermore, we hypothesize that tranilast, a clinically approved and inexpensive anti-fibrotic drug with decades of safe use can be repurposed to alleviate mechanical stress in solid tumors and that tranilast-induced stress alleviation can improve the delivery and efficacy of common anticancer drugs of all sizes. While not fully elucidated, the mechanism by which tranilast exerts its anti-fibrotic effects has been shown to be mediated, at least in part, via inhibition of TGFβ1 secretion, suppression of collagen biosynthesis as well as downregulation of connective tissue growth factor (CTGF)-induced extracellular matrix accumulation^32^,^33^,^34^. In this work, we first used computational modeling to validate if the general principle is mathematically consistent and to confirm the hypothesis that this effect is drug size-independent. Subsequently, to experimentally test our hypotheses, we employed two breast cancer cell lines (MCF10CA1a and 4T1) and three drugs of different sizes, namely doxorubicin (<1 nm), Abraxane (10 nm) and Doxil (100 nm) and showed that tranilast can improve the efficacy of all drugs by alleviating tumors’ solid and fluid stresses.

## Materials and Methods

### Cell culture

MCF10CA1a human breast cancer cell line was obtained from the Karmanos Cancer Institute (Detroit, MI, USA) and maintained as previously described^35^. 4T1 mouse mammary carcinoma cell line was purchased from ATCC and maintained in RPMI 1640 medium supplemented with 10% fetal bovine serum (FBS).

### Drugs and reagents

For *in vivo* studies, tranilast (Rizaben, Kissei Pharmaceutical, Japan) was solubilized with 1% NaHCO_3_ followed by heating at 70 °C for 1 h (33.3 mg/ml), as previously described^36^,^37^. For *in vitro* experiments, tranilast was dissolved with DMSO at 100 mM stock concentration and TGFβ1 (R&D systems) was dissolved with sterile 4 mM HCl containing 1 mg/ml BSA at 1 μg/ml stock concentration. Doxorubicin hydrochloride (Sigma) was dissolved (625 μg/ml stock) in phosphate buffer saline (PBS). Doxil (Pegylated liposomal doxorubicin, Janssen Pharmaceuticals) was purchased as already made solution (2 mg/ml) and Abraxane (Albumin-bound paclitaxel, Celgene) was solubilized in 0.9% NaCl in final stock concentration of 5 mg/ml.

### Animal tumor models and treatment protocols

Orthotopic xenograft breast tumors were generated by implantation of 5 × 10^5^ MCF10CA1a cells in 40 μl of serum-free medium into the mammary fat pad of 6-week old female CD1 nude immunodeficient mice. Orthotopic syngeneic models for murine mammary tumors were generated by implantation of 10^5^ 4T1 mouse mammary cancer cells in 40 μl of serum-free medium into the mammary fat pad of 6-week old BALB/c female mice. In both animal tumor models, tranilast (200 mg/kg) was administered orally once a day from day 4 post-implantation. Doxorubicin (2 mg/kg and 5 mg/kg for the MCF10CA1a and 4T1 models, respectively) was administered by intraperitoneal (i.p.) injection from day 11 post-implantation, every 72 hours. Doxil (3–6 mg/kg) and Abraxane (20 mg/kg) were administered by intravenous (i.v.) injection on days 14, 21 and 28 post-implantation^10^,^28^,^29^,^38^. During the course of each experiment, tumor growth was monitored daily and the planar dimensions (*x, y*) were measured using a digital caliper. Tumor volume was calculated using the volume of an ellipsoid and assuming that the third dimension, *z*, is equal to 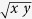. All *in vivo* experiments were conducted in accordance with the animal welfare regulations and guidelines of the Republic of Cyprus and the European Union (European Directive 2010/63/EE and Cyprus Legislation for the protection and welfare of animals, Laws 1994-2013) under a license acquired and approved (No CY/EXP/PR.L1/2014) by the Cyprus Veterinary Services committee, the Cyprus national authority for monitoring animal research for all academic institutions.

### Experimental procedure

To measure alterations in the tumor microenvironment, right before the end of the experiment, animals were anesthetized by i.p. injection of Avertin (200 mg/kg) and interstitial fluid pressure was measured using the wick-in-needle technique^23^,^39^,^40^. Next, mice were injected intracardially with 100 μl biotinylated lectin (1 mg/ml, Vector Labs) which was allowed to distribute throughout the body for 7 minutes^29^. Finally, mice were sacrificed via CO_2_ inhalation and tumors were excised for measurement of mechanical properties and/or histological analysis.

### Fluorescent immunohistochemistry and vessel perfusion histology

MCF10CA1a and 4T1 breast tumors were excised from mice, fixed and embedded in optimal cutting temperature compound (OCT). Transverse 40 μm-thick tumor sections were produced using the Tissue-Tek Cryo3 (SAKURA) and immunostained with antibodies against collagen I, CD31 and hyaluronan. For Ki67 staining, tumors were fixed in paraformaldehyde and embedded in paraffin before sectioning^35^.

For blood vessel perfusion analysis, mice were slowly injected with 100 μl of 1 mg/ml biotinylated lycopersicon esculentum lectin (Vector Labs) via intracardiac injection 7 minutes prior to euthanization and tumor removal. Upon excision, tumors were fixed in paraformaldehyde, embedded in OCT and frozen. Transverse 60 μm-thick tumor sections were produced and stained with an antibody CD31, streptavidin-conjugated and fluorescently-labeled secondary antibodies.

In addition, MCF10A1a tumors were immunostained with an anti-phosphorylated-Smad2 (Ser465/467)/Smad3 (Ser423/425) antibody and counterstained with anti-β-tubulin. Images from anti-collagen I, anti-CD31, anti-hyaluronan and anti-biotin-stained sections were analysed based on the area fraction of positive staining. To avoid any bias, the analysis was performed automatically using a previously developed in-house code in MATLAB (MathWorks, Inc., Natick, MA, USA)^23^. Five different sections per tumor (from the interior and the periphery) at × 10 magnification were taken and analyzed keeping the analysis settings and thresholds the same for all tumors. More details can be found in the Supplementary Material.

### Mechanical testing measurements for calculation of elastic modulus and hydraulic conductivity

The elastic modulus was calculated using an unconfined compression experimental protocol. Following tumor excision, tumor specimens 3 × 3 × 2 mm (length × width × thickness) were loaded on a high precision mechanical testing system (Instron, 5944, Norwood, MA, USA) and compressed to a final strain of 30% with a strain rate of 0.05 mm/min, the minimum rate the system can apply in order to avoid any transient, poroelastic effects. The elastic modulus was calculated from the slope of the stress-strain curve (details in Supplementary Material).

For the calculation of the hydraulic conductivity, stress relaxation experiments were performed in compression. Specimens underwent four cycles of testing for each of which a 5% compressive strain was applied for 1 minute, followed by a 10 minute hold and the stress vs. time response of the tissue was recorded. Subsequently, a common biphasic model of soft tissue mechanics was employed^41^ accounting for both the solid phase (cells and extracellular matrix) and the fluid phase (interstitial fluid) of the tumor. The hydraulic conductivity was calculated by fitting the model to the experimental stress-time data (Fig. S5, details in Supplementary Material).

A description of the mathematical model, IFP measurements, biodistribution analysis, *in vitro* cell culture experiments, gene expression analysis and statistical analysis can be found in the Supplementary Material and Supplementary Table S1.

## Results

### Mathematical modeling of stress alleviation mechanism

**Figure 1:**
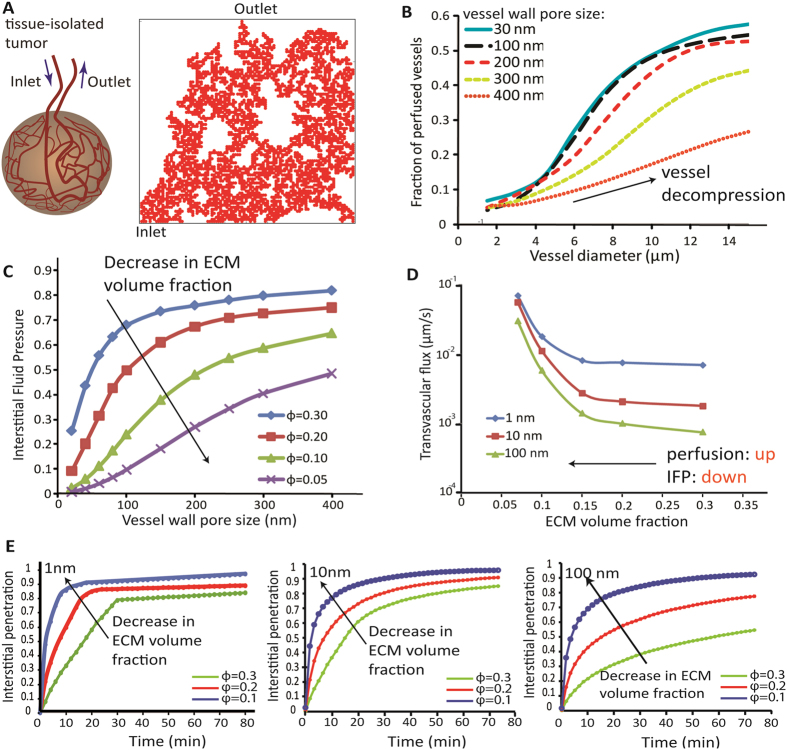
Mathematical model predictions of stress alleviation strategy. (**A**) A tissue isolated tumor consisted of one inlet and one outlet was modeled. The side length of the computational domain (right) is 1 cm. (**B**) Fraction of perfused vessels as a function of the vessel diameter for 5 different values of the vessel wall pore size. Perfused vessels were considered to be those with velocities greater than 0.1 mm/s. (**C**) Interstitial fluid pressure at the center of the tumor as a function of the vessel wall pore size for 4 different values of the ECM fiber volume fraction. (**D**) Transvascular flux (i.e., rate of nanoparticles crossing the tumor vessel wall) of nanoparticles of 3 distinct sizes as a function of ECM fiber volume fraction. (**E**) Interstitial penetration of nanoparticles of 1 nm, 10 nm and 100 nm in diameter as a function of time from entering the tumor vasculature and for three values of the ECM fiber volume fraction. Interstitial penetration was considered to be the area in the tumor covered by nanoparticles in concentrations above 5% of the peak concentration of the drug at the inlet of the tumor vasculature.

Initially, we performed mathematical modeling analysis to investigate the effect of stress alleviation strategy via ECM depletion on drug delivery. The purpose of using model simulations was to confirm whether the general principle indicating that stress alleviation can cause size-independent delivery of drugs is mathematically consistent. The tumor was modeled in two dimensions with its vasculature represented by a percolation network consisting of one inlet and one outlet (Fig. 1A)^10^,^42^. Each vascular node was assigned a vessel diameter and vessel wall pore size. We solved the equations of fluid flow and nanoparticle transport in the vascular, transvascular and interstitial space of the tumor and calculated blood vessel velocity, IFP and drug delivery. The first effect of stress alleviation is decompression of tumor blood vessels to improve perfusion. Vessel decompression was modeled as an increase in vessel diameter. Interestingly, an unexpected insight of the model is that the increase in the vessel diameter improves the fraction of perfused vessels independently of the vessel wall pore size (Fig. 1B) making this strategy suitable for both moderately as well as highly permeable tumors. Furthermore, depletion of ECM components increases the hydraulic conductivity - the ease with which interstitial fluid percolates through the interstitial space of the tumor^43^ (Supplementary Eq. S13) - which in turn reduces IFP (Fig. 1C). Therefore, contrary to vascular normalization, in stress alleviation therapy IFP reduction is achieved without affecting the pores of the tumor vessel wall (i.e., the EPR effect). Improved tumor perfusion and reduced IFP enhances drug transport from the vessels into the tumor interior (Fig. 1D). Finally, the creation of a larger interstitial space available for diffusion, owing to ECM depletion, improves the intratumoral penetration of small (1 nm, 10 nm) and large (100 nm) nanoparticles (Fig. 1E). Importantly, model predictions suggest that stress alleviation improves delivery of drugs of all sizes (Fig. 1D,E). The extent of improved delivery depends on the permeability of the tumor blood vessels but not on the lymphatic function at the tumor periphery (Supplementary Figs S1 and S2). Particularly, for 10 nm drugs and smaller, the benefit of stress alleviation appears to be optimal for tumors with moderately permeable vessels (Supplementary Fig. S1), such as pancreatic ductal adenocarcinomas, because for these tumors the effect of vessel decompression on perfusion and thus, on the functional vessel density is optimized (Fig. 1B). The delivery of larger particles, however, depends not only on the functionality of the tumor vessels but also on the ability of the particles to cross the tumor vessel wall into the tumor interior. Therefore, vessel decompression is expected to optimize the delivery of the 100 nm particles for tumors with highly permeable vessels (Supplementary Fig. S1), such as a subset of mammary carcinomas.

### Tranilast improves the efficacy of common anticancer drugs in a size-independent manner

**Figure 2:**
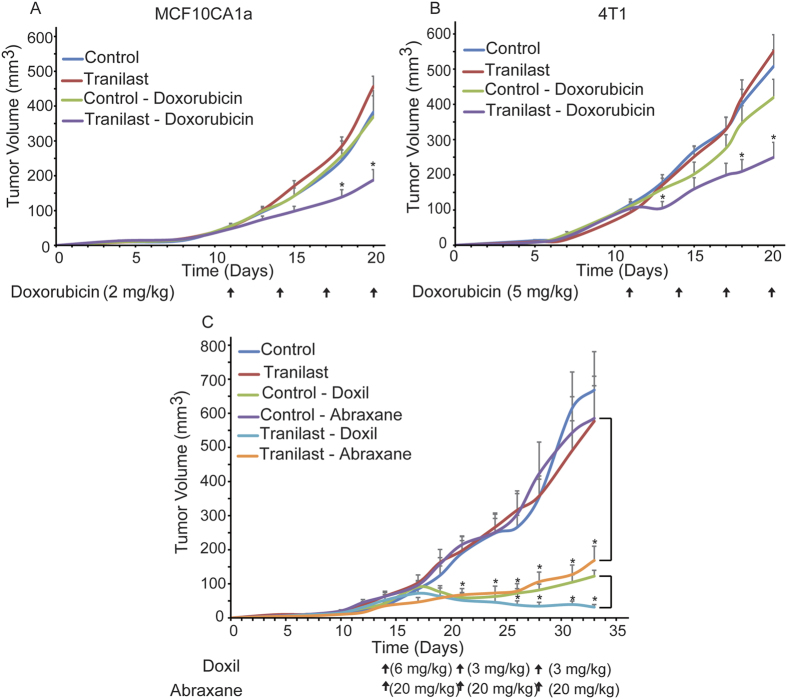
Tranilast increases anti-tumor efficacy of chemo- and nanotherapy. Tumor volume growth rates of orthotopic MCF10CA1a human breast tumors (**A**) and 4T1 murine breast tumors (**B**) implanted in female CD1 nude or BALB/c mice, respectively. Control treatment (NaHCO_3_ and saline), tranilast or doxorubicin alone had no effect on tumor growth in both tumor models. Combination of tranilast (200 mg/kg) and doxorubicin (2 mg/Kg) significantly delayed tumor growth of MCF10CA1a breast tumors compared to doxorubicin monotherapy (*p* = 0.01 on day 20, *n* = 6–8). Similarly, while doxorubicin alone (5 mg/kg) had no effect on growth of 4T1 murine breast tumors, combination of tranilast and doxorubicin significantly reduced tumor growth rate (*p* = 0.015 on day 20, *n* = 6–8). (**C**) Tumor volume growth rates of orthotopic MCF10CA1a breast tumors implanted in CD1 nude mice that were either control-treated (0.9% NaCl for Abraxane, saline for Doxil and NaHCO_3_ for tranilast), tranilast-treated alone (200 mg/kg daily via gavage), Doxil alone (6 mg/kg on day 14 and 3 mg/kg on days 21 and 28 post-implantation, i.v.), Abraxane alone (20 mg/kg on days 14, 21 and 28 post-implantation, i.v.), or tranilast in combination with either Doxil or Abraxane, as described above. Abraxane alone had no effect on tumor volume, compared to administration of tranilast with Abraxane, which significantly delayed tumor growth (*p* = 0.003 on day 33, *n* = 7–9). Doxil monotherapy at 6 mg/kg decreased tumor volume whereas at 3 mg/kg it only delayed tumor growth compared to control. On the other hand, combination of 3 mg/kg Doxil and tranilast significantly reduced tumor volume (*p* = 0.0001 on day 33, *n* = 7–9) compared to Doxil (3 mg/kg) alone. Asterisks indicate a statistically significant difference between compared groups (*p* < 0.05).

Based on these predictions, we hypothesized that tranilast can improve the efficacy of therapeutics via stress alleviation. To investigate our hypothesis, we developed two orthotopic mouse models for breast cancer; a xenograft model using the human MCF10CA1a cancer cell line and a syngeneic model using the mouse 4T1 cancer cell line and employed three commonly used drugs: doxorubicin, which has a size of less than 1 nm; Abraxane, a 130 nm albumin-bound paclitaxel that shrinks to 10 nm following dilution to plasma^10^; and Doxil, a ~100 nm pegylated liposomal doxorubicin. Mice received orally mock treatment or tranilast (200 mg/kg) 4 days post-implantation of cancer cells in the mammary fat pad, whereas anti-tumor drug administration begun on day 11. Higher doses of tranilast have been previously associated with direct anti-tumor and anti-vascular effects, whereas lower doses did not cause any effect on ECM composition^36^,^37^,^44^. We found that tranilast alone did not influence primary tumor growth (Fig. 2) or cell proliferation *in vivo* based on the number of Ki67 positive cells (Supplementary Figure S3). Similarly, doxorubicin (2 mg/kg to 5 mg/kg, i.p.) had no effect on tumor growth in either animal model. However, combinatorial treatment with tranilast and doxorubicin significantly delayed tumor growth (Fig. 2A,B and Supplementary Fig. S4A,B). Next, we investigated the role of tranilast in the efficacy of nanotherapeutics. Our data indicate that tranilast dramatically improved the efficacy of i.v. administered Abraxane (20 mg/kg) as depicted by the substantial differences in breast tumor growth rates (Fig. 2C, Supplementary Fig. S4C). Similarly, while initial i.v. administration of 6 mg/kg Doxil had a similar effect in control and tranilast-treated mice, lowering the dose (3 mg/kg) resulted in a differential response between the two groups (Fig. 2C). In agreement with model predictions, our data suggest that tranilast improves the efficacy of drugs in a size-independent manner.

### Tranilast remodels the tumor microenvironment reducing collagen and hyaluronan levels

**Figure 3:**
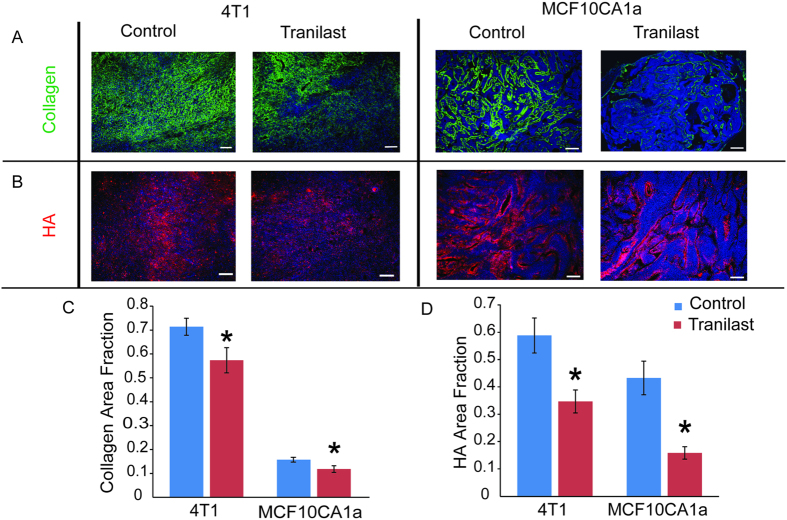
Tranilast decreases levels of extracellular matrix components. Representative images from histological analysis showing the effect of tranilast on reducing (**A**) collagen (green) and (**B**) hyaluronan (HA, red) levels in 4T1 and MCF10CA1a breast tumors. Area fractions of collagen (**C**) and hyaluronan (HA) (**D**) were significantly lower in tranilast-treated compared to control-treated tumors (*p* = 0.025, 4T1; *p* = 0.024, MCF10CA1a for collagen, *p* = 0.043, 4T1; *p* = 0.016, MCF10CA1a for hyaluronan, *n* = 6 from each group). Asterisks indicate statistically significant difference between groups. Scale bar: 100 μm.

To investigate the ability of tranilast to remodel the tumor ECM, we initially performed histological analysis of tumor cryosections. Tranilast treatment decreased the amount of both collagen and hyaluronan, as represented by area fraction quantification (Fig. 3). Specifically, area fractions of collagen (Fig. 3C) were reduced by 20% and 25% and of the hyaluronan (Fig. 3D) by 40% and 63% in the 4T1 and MCF10CA1a tranilast-treated tumors, respectively compared to control tumors (*p* = 0.025, 4T1; *p* = 0.024, MCF10CA1a for collagen and *p* = 0.043, 4T1; *p* = 0.016, MCF10CA1a for hyaluronan).

### Tranilast alleviates intratumoral solid and fluid stresses

**Figure 4:**
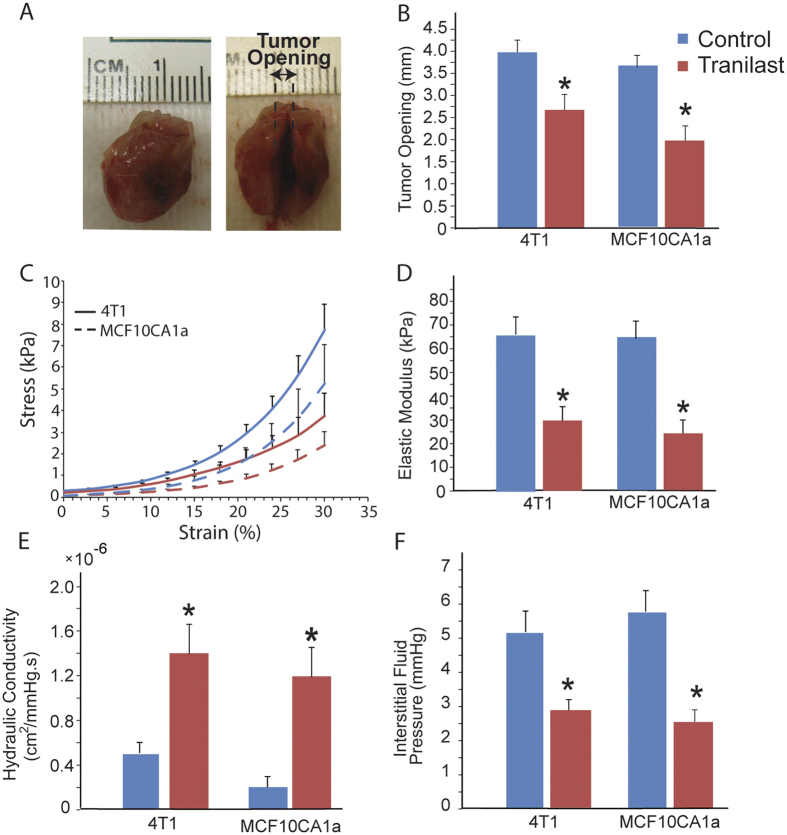
Tranilast alleviates solid and fluid stresses. (**A**) Representative images before and after a tumor opening experiment that was performed on a tranilast-treated tumor as a measure of growth-induce solid stress. After excision a cut was made ~80% of the thickness of control or tranilast-treated tumors along the main axis and the tumor opens up as a result of stress release. (**B**). Sample-blind measurements of tumor opening for the two different cancer cell lines indicate that treatment with tranilast leads to lower values of relaxation compared to control treatment (*p* = 0.013 for 4T1, *p* = 0.005 for MCF10CA1a), resulting to lower levels of growth-induced stress. (**C**) Stress-strain curves (means and standard errors) of the two cancer cell lines for the control (blue) and tranilast-treated (red) groups. (**D**) The elastic modulus of the two cancer cell lines measured from the slope of the stress-strain curves at 30% strain. The values of the elastic modulus for the tranilast-treated tumors are significantly lower (*p* = 0.002 for 4T1, *p* = 0.041 for MCF10CA1a). Tranilast-induced reduction in ECM content resulted to lower values of the (**E**) hydraulic conductivity of the tumor interstitial space (*p* = 0.015 for 4T1, *p* = 0.029 for MCF10CA1a), which in turn caused alleviation of the (**F**) interstitial fluid pressure (*p* = 0.013 for 4T1 and *p* = 0.012 for MCF10CA1a).

Subsequently, to further explore the potential of tranilast as a stress-alleviating agent, we performed detailed analysis of the mechanical properties of the tumors. We first measured the growth-induced stresses by employing our previously developed technique, the tumor opening experiment^23^. Upon excision, we cut the tumors along their longest axis at approximately 80% of their thickness. We then allowed tumors to relax and measured the formed distance between the two hemispheres (Fig. 4A). Control tumors had significantly larger tumor openings, which corresponds to higher growth-induced solid stress compared to tranilast-treated tumors (Fig. 4B). We also performed *ex-vivo* stress-strain experiments, which revealed that tranilast decreased the elastic modulus of the tumors, making them less stiff (Fig. 4C,D). Collectively, our data from the tumor opening and stress-strain experiments clearly suggest that tranilast decreases solid stresses (i.e., stresses of the solid phase) in both MCF10CA1a and 4T1 tumor models. In addition, we examined the effect of tranilast on tumor fluid phase features and particularly on the interstitial hydraulic conductivity and fluid pressure. To calculate the hydraulic conductivity, we performed *ex vivo* stress-relaxation experiments and the data were fitted to a biomechanical mathematical model (Supplementary Fig. S5). Treatment with tranilast increased hydraulic conductivity (Fig. 4E), which was expected due to the reduction in ECM components (Supplementary Eq. S13). According to our mathematical analysis (Fig. 1C), the increase in the hydraulic conductivity should cause alleviation of the IFP. Consistent to our predictions, using the wick-in-needle technique^39^, we found that tranilast significantly decreased IFP in both tumor models (Fig. 4F).

### Alleviation of stresses decompresses tumor blood vessels and improves perfusion

**Figure 5:**
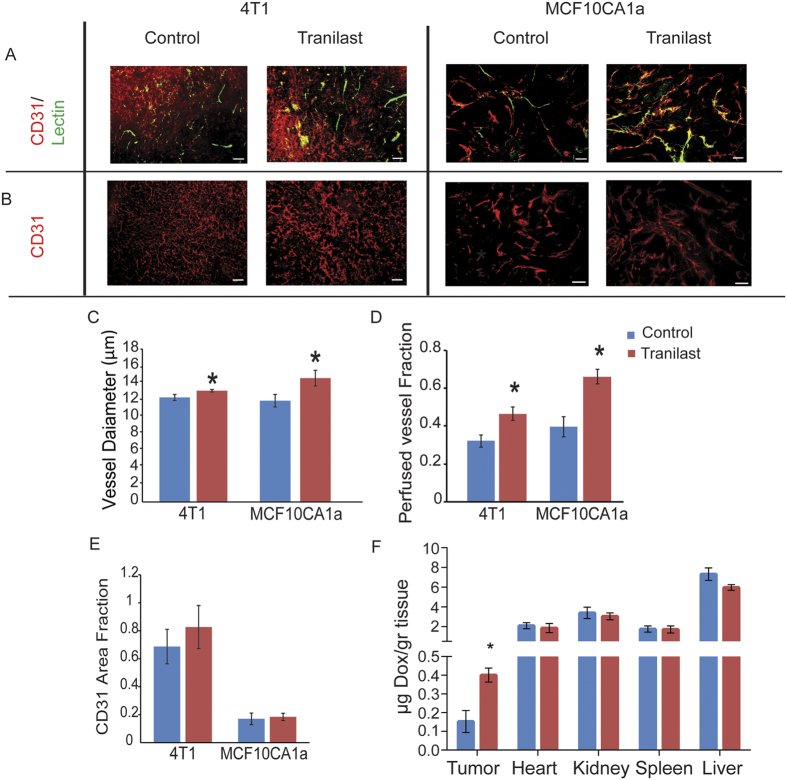
Tranilast-induced stress alleviation decompresses tumor blood vessel improving perfusion and doxorubicin delivery in tumors. Representative images from histological analysis (**A**). Endothelial cell-specific marker CD31 (red), biotinylated tomato lectin (green) and co-expression (yellow) indicating the presence of perfused vessels, and (**B**). Expression of CD31 (red) indicating blood vessels in 4T1 and MCF10CA1a tumors. Quantification of blood vessel diameter (**C**), fraction of perfused vessels (**D**), and CD31 (**E**) area fractions in control and tranilast-treated tumors. Vessel diameter was significantly increased in both tumor models (*p* = 0.038, 4T1 and *p* = 0.04, MCF10CA1a, *n* = 6–8), resulting in a significant increase in the perfused vessel fraction (*p* = 0.027, 4T1 and *p* < 0.001, MCF10CA1a, *n* = 6–8), whereas CD31 area fraction was unaffected by tranilast in both tumor models. Asterisks indicate a statistically significant difference between compared groups. Scale bar: 100 μm. (**F**). Quantification of doxorubicin concentration in control versus tranilast-treated MCF10CA1a breast tumors, liver, heart and spleen tissues from corresponding CD1 nude mice. Doxorubicin (225 μg) was administered by intravenously (i.v.) via tail vein injection to the animals 4 hours prior to sacrifice. Doxorubicin concentration in tissue sample homogenates was determined by quantification of fluorescence intensity (Ex.: 470 nm, Em: 590 nm). Data represent the average + S.E. values and (*) indicates statistically significant differences between compared groups (n = 7, p < 0.05). Tranilast improved the delivery of doxorubicin in tumors by 2.5 times (p = 0.005).

To investigate the effect of stress alleviation on the functionality of tumor blood vessels, we calculated the vessel diameter and the percentage of perfused blood vessels after mock or tranilast treatment, respectively. Tissue cryosections were stained with antibodies against CD31 and biotinylated lectin. Based on the ratio of the number of biotinylated lectin positive (+) to the CD31 positive (+) vessels, the fraction of perfused vessels was calculated. Tranilast-induced stress alleviation resulted in a statistically significant increase in vessel diameter for both tumor models, which in turn caused a significant increase in tumor perfusion (Fig. 5A–D). Interestingly, despite the small increase in vessel diameter by 10–15%, the fraction of perfused vessels increased significantly by 50–60%. This is in full agreement with our previous studies^23^,^29^ and could be explained by the fact that blood flow rate is proportional to the fourth power of the vessel diameter (Supplementary Eq. S1) so that small increases in diameter could improve significantly blood flow. Furthermore, it could be also possible a compressed upstream vessel to exclude from blood flow a large number of downstream vessels, which will become functional when the upstream vessel is decompressed. In contrast, the total area of vessels (CD31 + area) remained unaffected (Fig. 5E), suggesting that tranilast treatment improved perfusion without affecting tumor angiogenesis. Importantly, *in vivo* biodistribution analysis revealed that improved tumor perfusion via stress alleviation resulted in a significant increase in intratumoral drug delivery without affecting delivery in normal tissues (Fig. 5F).

### Tranilast inhibits TGFβ pathway and downregulates COL1A1, CTGF and HAS gene expression

**Figure 6:**
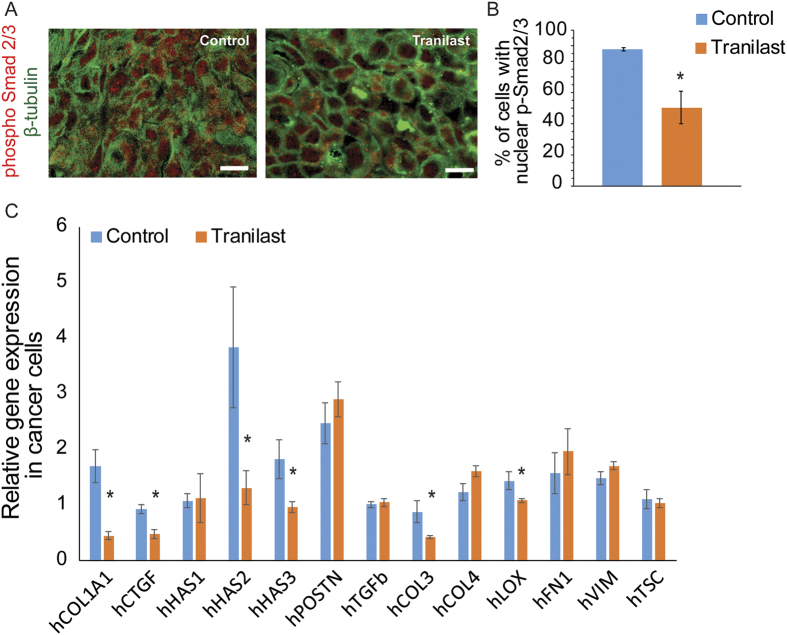
Tranilast suppresses human COL1A1, CTGF, HAS2, HAS3, COL3 and LOX gene expression as well as Smad2/3 phosphorylation and nuclear translocation *in vivo*. (**A**) Representative images of MCF10CA1a tumor sections and immunofluorescent staining using confocal microscopy. Tumor sections from control-treated or tranilast-mice were stained with anti-phospho-Smad2/3 (red) antibody and counterstained with anti-β-tubulin (green) antibody for detection of the cytoskeleton, indicated that tranilast inhibits Smad2/3 phosphorylation and nuclear translocation. Scale bar: 100 μm. (**B**) Quantification of Smad2/3 phosphorylation and nuclear translocation was performed by counting of the p-Smad2/3-stained nuclei (red) and expressed as percentage of red positive nuclei compared to the total number of cells using confocal images from 3 independent experiments. (**C**) Real-time PCR gene expression analysis and quantification of hCOL1A1, connective tissue growth factor hCTGF, hHAS1, hHAS2, hHAS3, hPOSTN, hTGFβ, hCOL3A1, hCOL4A1, hLOX, hFN1, hVIM and hTSC mRNA levels extracted from control-treated compared to tranilast-treated MCF10CA1a tumors, indicated that tranilast suppresses COL1A1, CTGF, HAS2 and HAS3 gene expression. Relative expression for all genes in both groups was normalized based on the expression of beta-actin. Data represent the average of at least 3 independent experiments from 5 control and 5 tranilast-treated tumors ± S.E. values and asterisks indicate statistically significant differences between compared groups (*p* < 0.05).

Finally, based on previous work related to the biological mechanisms underlying tranilast’s mode of action^32^,^33^,^34^, we wanted to investigate whether similar effects could also be exerted during tranilast-induced remodeling of the tumor microenvironment. To elucidate these molecular events, we first performed immunofluorescence analysis in MCF10CA1a tumors, which indicated that tranilast suppressed Smad2/3 phosphorylation and nuclear translocation *in vivo* (Fig. 6A), suggesting that inhibition of TGFβ signaling pathway could be responsible, at least in part, for the observed reduction of extracellular matrix components, stress alleviation and increased drug delivery in tumors. We then quantified the *in vivo* expression of critical TGFβ target genes that are involved in collagen and hyaluronan synthesis as well as other encoding for extracellular matrix components or collagen crosslinking enzymes. RNA extraction from human MCF10CA1a tumors followed by real-time PCR using human-specific primers showed that tranilast suppressed collagen I (COL1A1), connective tissue growth factor (CTGF), hyaluronan synthase 2 (HAS2), hyaluronan synthase 3 (HAS3), collagen 3 (COL3A1) and lysyl oxidase (LOX) gene expression in breast cancer cells (Fig. 6B). To investigate the effects of tranilast on tumor stromal cells, which include mouse cancer-associated fibroblasts and immune cells, we designed mouse-specific primers and performed real-time PCR analysis. We found that while tranilast did not have a major effect on most genes of the stromal cells, it suppressed periostin (POSTN) and induced hyaluronan synthase 3 expression (Suppl. Fig. S6). To further confirm that these genes are regulated by the TGFβ pathway, we performed *in vitro* cell culture experiments which showed that while TGFβ is able to activate COL1A1, CTGF, HAS1 and HAS2 gene expression in 4T1 mammary adenocarcinoma cells, pre-treatment with tranilast significantly inhibited TGFβ-mediated upregulation of COL1A1, CTGF and HAS2, but not HAS1 (Supplementary Fig. S7). Finally, since tranilast can directly alter gene expression, we wanted to investigate the possibility whether gene expression changes could also alter the sensitivity of the cancer cells to doxorubicin. Therefore, we performed an *in vitro* assay to calculate the percentage of viable 4T1 breast cancer cells that were treated either with tranilast alone, doxorubicin alone or combination of tranilast with doxorubicin. These data indicated that while doxorubicin alone significantly reduced breast cancer cell viability, combination of tranilast with doxorubicin did not further decrease the number of viable breast cancer cells (Supplementary Fig. S8). Conclusively, our *in vitro* and *in vivo* evidence suggest that tranilast primarily enhances drug delivery and efficacy of doxorubicin by remodeling the tumor microenvironment, whereas it does not seem to significantly sensitize cancer cells to chemotherapy.

## Discussion

To conclude, the objective of our study was two-fold. The first objective was to show the potential of stress alleviation treatment to improve the efficacy of chemo- and nano-therapeutics. Stress alleviation is a recently developed therapeutic strategy, which has shown promising results in improving chemotherapy in preclinical tumor models^29^,^30^,^31^,^45^,^46^,^47^ but there was no mathematical/theoretical basis for the effects of this strategy and also there was no study to explore its potential use for nanomedicines, where compromised drug delivery is a major barrier to their efficacy. Here, we demonstrated both mathematically as well as experimentally that stress alleviation improves the efficacy of common chemotherapeutics and nanomedicines in a size-independent manner. The second objective was to introduce a safe, well-tolerated drug as a stress-alleviating agent. Tranilast is an inexpensive, clinically approved drug that has already been tested in humans for toxicity and tolerance, rendering the results of our study highly transferable to the clinic. Therefore, tranilast-induced stress alleviation combined with common anticancer drugs could directly lead to Phase II clinical trials to test the efficacy of this therapeutic strategy in humans.

Stress alleviation strategy is expected to be beneficial for tumors that have abundant compressed vessels. Vessel compression in turn is caused by mechanical stresses exerted by the cells and the extracellular matrix. Therefore, desmoplastic tumors (e.g. pancreatic and breast cancers as well as sarcomas), which contain high levels of stromal cells and ECM components should have a large amount of compressed vessels. In principle, judicious depletion of either stromal cells or ECM components or both will have the ability to alleviate stresses in order to decompress blood vessels and improve perfusion in these tumors^23^,^24^,^25^. Furthermore, as shown here, stress alleviation strategy is beneficial even for tumors with hyper-permeable vessels (e.g. a subset of breast cancers) where perfusion depends not only on vessel diameter but also on vessel permeability. In the case of tumors with compressed and hyper-permeable vessels, stress alleviation strategy could be combined with vascular normalization so that blood vessels will become decompressed and less permeable, which should optimize perfusion and delivery of small-sized drugs (Fig. S1)^48^. The degree of vessel compression and permeability, however, can vary considerably not only among tumor types but also within the same tumor type as well as between the primary tumor and its metastases. Therefore, it is difficult to choose an appropriate strategy until the state of that individual tumor is known. Emerging imaging approaches have the potential to help in this selection^49^.

Furthermore, it is reasonable to argue that improving tumor perfusion could also allow more oxygen and nutrients to be transported in the tumor and thus, increase its growth rate. Additionally, the decompressed vessels could potentially assist metastatic cells to leave the primary tumor and form metastases, which has been shown in pre-clinical studies^50^,^51^. Therefore, drugs that decompress vessels should only be given with concurrent cytotoxic treatments, such as chemotherapy, nanomedicine, radiation therapy or immune therapy. Specifically, as far as the use of tranilast is concerned, preclinical studies have indicated that it does not enhance metastatic dissemination^36^,^37^,^44^.

Finally, even though our mathematical model is general and the mechanism of tranilast action was not explicitly incorporated, this could be feasible through its involvement in the TGFβ pathway and could be used for the investigation of further interactions between TGFβ and tumor perfusion that go beyond the scope of the current study.

## Additional Information

**How to cite this article:** Papageorgis, P. *et al*. Tranilast-induced stress alleviation in solid tumors improves the efficacy of chemo- and nanotherapeutics in a size-independent manner. *Sci. Rep.* **7**, 46140; doi: 10.1038/srep46140 (2017).

**Publisher's note:** Springer Nature remains neutral with regard to jurisdictional claims in published maps and institutional affiliations.

## Supplementary Material

Supplementary Information
