# Supplementary material for: Tranilast-induced stress alleviation in solid tumors improves the efficacy of chemo- and nanotherapeutics in a size-independent manner
Source: Sci Rep. 2017 Apr 10;7:46140. doi: 10.1038/srep46140 (PMC5385877; doi:10.1038/srep46140)
Supplement: Supplementary Information [file srep46140-s1.pdf]

Supplementary Material

Tranilast-induced stress alleviation in solid tumors improves the efficacy of chemo- and nanotherapeutics in a size-independent manner

Panagiotis Papageorgis, Christiana Polydorou, Fotios Mpekris, Chrysovalantis Voutouri, Eliana Agathokleous, Constantina P. Kapnissi-Christodoulou and Triantafyllos Stylianopoulos

Description of mathematical model for nanoparticle delivery

The general mathematical framework (equations and solution strategy) of the model was developed in (1) and the model has been specified for the needs of the current study. To simulate drug delivery in tumors, we employed a mathematical model of fluid and mass transport in the vascular, transvascular and interstitial space. In previous research, we validated our model predictions for the delivery of nano-scale drugs to solid tumors with experimental data[1]. We modeled an isolated tumor consisting of one inlet and one outlet as shown in Suppl. Fig. 1A. To represent the tumor vasculature, we used an invasion-percolation network, which has been shown to have similar structural characteristics with the tumor vasculature [2-5].

In the vascular space the flow was assumed to follow Poiseuille's law and the drug transport was governed by convection:

$$Q_v = -\frac{\pi d^4}{128\mu}\nabla P_v \tag{S1}$$

$$\frac{dc_v}{dt} = -v\frac{\Delta c_v}{\Delta x} \tag{S2}$$

where Q_v is the blood flow rate, P_v is the vascular pressure, d the vessel diameter, μ the viscosity. In Eq. S2, v is the fluid velocity, which is determined by dividing Q_v in Eq. (S1) by the cross-sectional area of the vessel, c_v the vascular concentration of the drug and Δc_v is the concentration difference that corresponds to a vascular length Δx.

The transport across the tumor vessel wall was represented by Starling's approximation as:

$$Q_t = L_p S(P_v - P_i) \tag{S3}$$

$$\Phi = L_p S(P_v - P_i)(1 - \sigma)\frac{c_v e^{Pe} - c_i}{e^{Pe} - 1} \tag{S4}$$

where Q_t is the transvascular fluid flow rate, L_p is the hydraulic conductivity of the vessel wall, S the surface area of the wall, P_i the interstitial fluid pressure, Φ the drug flux across the vessel wall, σ the reflection coefficient, c_i the interstitial concentration of the drug and Pe is the Péclet number given as:

$$P_e = L_p(1 - \sigma)\frac{(P_v - P_i)}{P} \tag{S5}$$

with P the vascular permeability of the nanoparticle. The reflection coefficient describes the easiness by which a particle can be transported through the pores of the vessel wall and depends in our model on the relative size of the particle to the pore. If the particle's size is the same or larger than that of the pore of the vessel wall, then σ=1 and the particle cannot cross the wall. The smaller the particle compared to the pores the easier its transport becomes and σ approaches to zero.

Assuming the pores of the vessel wall to be of cylindrical shape, the hydraulic conductivity, L_p, vascular permeability, P, and reflection coefficient, σ, can be calculated as [6]:

$$L_p = \frac{\gamma r_o^2}{8\mu L} \tag{S6}$$

$$P = \frac{\gamma H D_o}{L} \tag{S7}$$

$$\sigma = 1 - W \tag{S8}$$

where γ is the fraction of wall surface area occupied by pores, r_o is the vessel wall pore radius (d_p/2), L is the thickness of the vessel wall, and D_o is the diffusion coefficient of the particle in free solution at 37°C calculated by the Stokes-Einstein relationship. The parameters H and W account for the hindrance in the transport of the nanoparticles due to hydrodynamic and electrostatic interactions with the vessel wall and their calculation is described in Ref. [6] as a function of particle size to vessel wall pore size. Therefore, assigning a pore size distribution in the model we can calculate these parameters.

Interstitial fluid flow is given by Darcy's law and interstitial mass transport can be both convective and diffusive. Thus,

$$Q_i = -K_t A_c \nabla P_i \tag{S9}$$

$$\frac{dc_i}{dt} = D\nabla^2 c_i - v_i \nabla c_i \tag{S10}$$

where K_t is the hydraulic conductivity of the interstitial space, A_c is the tissue cross-sectional area, D the diffusion coefficient of the nanoparticle, and v_i the interstitial fluid velocity given by Darcy's law. The tissue cross-sectional area is related to the vascular density, S_v, and the diameter of the vessel, d, [7]:

$$A_c = \frac{\pi d}{S_v} \tag{S11}$$

Solution of Governing Equations

The vascular and interstitial space were discretized with computational nodes. Each of the computational nodes that belongs to the vascular space was assigned a pore diameter and the values of the parameters L_p, P, and σ were calculated. The pore diameter was taken from a unimodal distribution based on experimental studies [8,9].

The fluid transport problem was solved first for the steady state and the vascular and interstitial fluid pressures were calculated. Conservation of the fluid requires that at each computational node the volume of fluid entering the node is the same as the volume exiting the node, i.e., $\sum_k Q_k = 0$ for each node k, either vascular or interstitial. As for boundary conditions, the vascular pressure at the inlet and outlet of the network was predefined. The boundaries of the interstitial space, were assumed to be surrounded by fully functional lymphatic vessels and thus the fluid pressure was zero.

The nanoparticle transport problem is transient and solved with a finite difference scheme. Central differencing for diffusion, upwind differencing for convection and explicit Euler time integration were used. The concentration of the particles at the inlet was specified and assumed to decay exponentially (i.e., bolus injection) with a given constant, i.e., $c_v = c_o e^{-t/K_d}$, K_d is the time constant for concentration decay in the plasma. Whereas, the concentration at the outlet and at the boundary of the interstitial space were set to zero.

Model Specification

Neutral nanoparticles of three different sizes, 10, 60 and 120 nm diameter were considered. The diffusion coefficient of the particles in the interstitial space of the tumor, D, was given by the product of hydrodynamic, F, and steric, S, interactions, as [10]:

$$\frac{D}{D_o} = F \cdot S = e^{-a\phi^b} \cdot e^{-0.84 f^{1.09}} \tag{S12}$$

where *D/Do* is the diffusion hindrance, i.e., the ratio of the diffusivity in the tumor to that in solution, and f is an adjusted volume fraction given as a function of the ECM volume fraction, φ, and the ratio of the solute diameter to the fiber diameter, λ (i.e., $f = (1+\lambda)^2\varphi$). The expressions for *F* and *S* were derived by Clague and Phillips [11], and Johansson and Lofroth [12], respectively, while values of *a* and *b* are given in Amsden [13] as: $a = \pi$, and $b = 0.174 ln(59.6/\lambda)$.

To relate the hydraulic conductivity of the interstitial space to modifications of the fiber volume fraction, we used published experimental data of the highly desmoplastic HSTS26T soft tissue sarcoma. The fiber volume fraction of this tumor model was estimated to be ~0.3 and the hydraulic conductivity was measured to be 0.9×10^{-7} $cm^2/mmHg\text{-}sec$ [14,15]. We used these values of fiber volume fraction and hydraulic conductivity for our baseline simulations. To estimate the effect of extracellular fiber depletion on the hydraulic conductivity we used a relationship that was developed for the hydraulic permeability, k ($k = \mu K_t$) of three-dimensional isotropic fibrous media [16]

$$\frac{k}{r^2} = \frac{3}{20\varphi}\left[-\ln(\varphi) - 0.931\right] \tag{S13}$$

where r is the radius of the fiber and φ the fiber volume fraction. Therefore the hydraulic conductivity after a decrease in the fiber volume fraction from $\varphi_1=0.3$ to φ_2 was estimated as

$$\frac{K_{t1}}{K_{t2}} = \frac{\varphi_2[-\ln(\varphi_1) - 0.931]}{\varphi_1[-\ln(\varphi_2) - 0.931]} \tag{S14}$$

Values for the model parameters are provided in Supplementary Table 1.

Fluorescent immunohistochemistry and vessel perfusion histology

MCF10CA1a and 4T1 breast tumors were excised from mice and fixed with 4% paraformaldehyde in PBS for 20 minutes at room temperature (RT), followed by overnight incubation at 30% sucrose in PBS at 4°C. The samples were then embedded in optimal cutting temperature (OCT) compound (Tissue-Tek) and frozen. Transverse 40μm-thick tumor sections were produced using the Tissue-Tek Cryo3 (SAKURA) and immunostained with collagen I antibody (ab4710, Abcam 1:100 dilution), with antibodies against the endothelial marker CD31 (MEC13.3, BD Biosciences, 1:100 dilution) and hyaluronan (ab53842, Abcam 1:100 dilution) counterstained with 4',6-diamidino-2-phenylindole (Vector Labs). Collagen I was detected with Alexa Fluor-647 Goat Anti-Rabbit IgG (H+L) secondary antibody (A-21244, Invitrogen, 1:800 dilution), CD31 with Alexa Fluor-647 Goat Anti-Rat IgG (H+L) secondary antibody (A-21247, Invitrogen, 1:800 dilution), and hyaluronan using Alexa Fluor-647 donkey Anti-sheep IgG (H+L) secondary antibody (A-11015, 1:800 dilution). For Ki67 staining, which marks the proliferating cells, breast tumors were post-fixed in 4% paraformaldehyde in PBS, dehydrated through ethanol series and embedded in paraffin. Transverse 7 μm-thick tumor sections were produced using the microtome (Accu-Cut SRM 200 Rotary Microtome, SAKURA). Deparaffinized sections underwent antigen retrieval (microwave heat treatment with TriSodium Citrate, pH 6, for 10 min). For detection of Ki67 protein, sections were incubated with a primary anti-Ki67 (M7248, DAKO ,1:50 dilution), followed by incubation with Alexa Fluor-647 Goat Anti-Rat IgG (H+L) secondary antibody (A-21247, Invitrogen, 1:400 dilution).

For blood vessel perfusion analysis, mice were slowly injected with 100 μl of 1 mg/ml biotinylated lycopersicon esculentum (tomato) lectin (Vector Labs) via intracardiac injection 7 minutes prior to euthanization and tumor removal. Upon excision, tumors were fixed in 4%

paraformaldehyde in PBS for 20 minutes at RT, followed by overnight incubation in 30% sucrose in PBS at 4°C. The samples were then embedded in OCT compound and frozen. Transverse 60 μm-thick tumor sections were produced and counterstained with antibody against the endothelial marker CD31 (MEC13.3 antibody, Biosciences (BD), 1:100 dilution). Lectin staining was detected using the Streptavidin Alexa Fluor 488 conjugate (Molecular probes, S11223, 1:400 dilution) and CD31 signal was detected with Alexa Fluor-647 Goat Anti-Rat IgG (H+L) secondary antibody (Molecular probes, A-21247, 1:800 dilution).

In addition, MCF10A1a tumors were immunostained with phosphorylated (P)-Smad2 (Ser465/467)/Smad3 (Ser423/425) Rabbit mAb (Cell Signaling D27F4, 1:100 dilution) and counterstained with β-tubulin (monoclonal anti-tubulin (E7) obtained from the Developmental Studies Hybridoma Bank, 1:100 dilution). The signal was detected using Alexa Fluor-647 Goat Anti-Rabbit IgG (H+L) A-21244 (1:400 dilution) and CF488A Donkey Anti-Mouse IgG (H+L) (20014-1, 1:400 dilution) secondary antibodies.

Images from collagen I, hyaluronan and CD31 stained sections were analysed based on the area fraction of positive staining using an in-house code in MATLAB (MathWorks, Inc., Natick, MA, USA) [17]. Vessel diameter was calculated from the CD31 images. Specifically, the procedure of the identification of the non-collapsed vessels was automated in the MATLAB code. The CD31 staining that forms a loop was considered to be a vessel and the short axis of a cross-section fit to an oval was taken. Five different sections per tumor (from the interior and the periphery) at ×10 magnification were taken and analysed keeping the analysis settings and thresholds the same for all tumors.

Biodistribution analysis

For biodistribution study a dose of 225μg of doxorubicin was injected via tail vein injection to the animals and 4 hours post injection they were sacrificed. Seven animals per group were tested (n=7). The tissues were excised from the mice and were stored at -80 °C until extraction. For doxorubicin extraction we used a previously described method with modifications [18]. The tissue samples were homogenized by using a homogenizer in 4 parts (v/w) cell lysis buffer (RIPA buffer). Then in 200μl of the homogenate were added 50μl 10% Triton X-100 and 750 μl 0.75N hydrochloric acid (HCl) in isopropanol. The mixture was well-vortexed and let overnight at -20°C for doxorubicin extraction. Samples were again vortexed at RT followed by centrifugation for 30 min at 4°C (14000 rpm). The supernatant was then collected from all samples and fluorescence was measured (Ex.: 470nm, Em: 590nm) using the Qubit 3.0 Fluorometer (Life Technologies). A standard curve was established by adding known amounts of doxorubicin to homogenates of non-treated tissue samples prior to extraction.

***In vitro* cell culture experiments.**

4T1 cells ($2x10^5$) were seeded in 6-well plates and the following day were pre-treated with 300μM Tranilast or DMSO (control) for 3 hours. Cells were then either mock-treated or treated with 1ng TGFβ for 24h followed by total RNA extraction and quantification of gene expression.

RNA isolation, cDNA synthesis, and real-time polymerase chain reaction:

Total RNA was isolated from breast tumors or cell lines using standard Trizol-based protocol (Invitrogen) and cDNA synthesis was performed using reverse transcriptase III (RT-III) enzyme and random hexamers (Invitrogen), as previously described[19]. Real-time polymerase chain reaction (PCR) was performed using Sybr Fast Universal Master Mix (Kapa). The following

mouse-specific primers were used for gene expression analysis of 4T1 mouse mammary carcinoma cells as well as stromal cells from MCF10CA1a tumors, while human-specific primers were used for gene expression analysis of cancer cells from MCF10CA1a tumors.

**Gene**	**Primer sequence**
**mCOL1A1 F**	GAGCGGAGAGTACTGGATCG
**mCOL1A1 R**	GTTCGGGCTGATGTACCAGT
**hCOL1A1 F**	GTGCTAAAGGTGCCAATGGT
**hCOL1A1 R**	ACCAGGTTCACCGCTGTTAC
**mCTGF F**	CACTCTGCCAGTGGAGTTCA
**mCTGF R**	GTAATGGCAGGCACAGGTCT
**hCTGF F**	CCGTACTCCCAAAATCTCCA
**hCTGF R**	ATGTCTTCATGCTGGTGCAG
**mHAS1 F**	TCGGAGATTCGGTGGACTAC
**mHAS1 R**	GTCCAACCTTGTGTCCGAGT
**hHAS1 F**	TCGGAGATTCGGTGGACTAC
**hHAS1 R**	GTCCAACCTTGTGTCCGAGT
**mHAS2 F**	ATAAGCGGTCCTCTGGGAAT
**mHAS2 R**	CCTGTTGGTAAGGTGCCTGT
**hHAS2 F**	ACCGGGGTAAAATTTGGAAC
**hHAS2 R**	TAAGGCAGCTGGCAAAAGAT

**mHAS3 F**	TTCCAAACCTCAAGGTGGTC
**mHAS3 R**	TGCTACGCCACACAAAGAAG
**hHAS3 F**	TTTGCCATTGCTACCATCAA
**hHAS3 R**	AGGCCAATGAAGTTCACCAC
**hB-ACTIN F**	CGAGCACAGAGCCTCGCCTTTGCC
**hB-ACTIN R**	TGTCGACGACGAGCGCGGCGATAT
**mB-ACTIN F**	GACGGCCAGGTCATCACTAT
**mB-ACTIN R**	AAGGAAGGCTGGAAAAGAGC
**mTSC F**	TGGAGTACGAGCTGCATGAC
**mTSC R**	AAACTTGGTGGCGATGGTAG
**mPOSTN F**	AGTTTGTTCGTGGCAGCAC
**mPOSTN R**	GAAGTCGGGATCACCTTCAA
**mVIM F**	ACAAAATCCTGCTGGCTGAG
**mVIM R**	CTTGTCGTTGGTGAGCTGAT
**hFN1 F**	GATGCTCCCACTAACCTCCA
**hFN1 R**	CGGTCAGTCGGTATCCTGTT
**hTSC F**	AACAAGCCACAACCAAAACC
**hTSC R**	GCTGGATTGCTCTCCTTGTC
**hPOSTN F**	TTCTGACGCCTCAAAACTGA
**hPOSTN R**	TGCTCTCCAAACCTCTACGG
**hVIM F**	CGAAAACACCCTGCAATCTT
**hVIM R**	ATTCCACTTTGCGTTCAAGG
**hCOL3 F**	TAGGTCCATCTGGTCCTGCT

**hCOL3 R**	CGAAGCCTCTGTGTCCTTTC
**hCOL4 F**	CTCTACGTGCAAGGCAATGA
**hCOL4 R**	AGAACAGGAAGGGCATTGTG
**mFN1 F**	CCCAGTGATTTCAGCAAAGG
**mFN1 R**	CCCAGTGATTTCAGCAAAGG
**mTSC F**	TGGAGTACGAGCTGCATGAC
**mTSC R**	AAACTTGGTGGCGATGGTAG
**mPOSTN F**	AGTTTGTTCGTGGCAGCAC
**mPOSTN R**	GAAGTCGGGATCACCTTCAA
**mVIM F**	ACAAAATCCTGCTGGCTGAG
**mVIM R**	CTTGTCGTTGGTGAGCTGAT
**mCOL3A1 F**	ATAAGCCCTGATGGTTCTCG
**mCOL3A1 R**	GCAGCCTTGGTTAGGATCAA
**mCOL4A1 F**	CCAAAGGATCAGTTGGAGGA
**mCOL4A1 R**	CTCCTTTGGCTCCCTTCTCT
**mLOX F**	GCACACACACAGGGATTGAG
**mLOX R**	CCAGGTAGCTGGGGTTTACA
**hLOX F**	CAGAGGAGAGTGGCTGAAGG
**hLOX R**	CCAGGACTCAATCCCTGTGT

Reactions were performed using a CFX-96 real-time PCR detection system (Biorad) using the following conditions: 95°C for 2 min, 95°C for 2 sec, 60°C for 20 sec, 60°C for 1 sec, steps 2-4 for 39 cycles.

Real-time PCR analysis and calculation of changes in gene expression between compared groups was performed using the ΔΔCt method, as previously described [19].

Unconfined compression experiments for calculation of elastic modulus and hydraulic conductivity

Stress-strain experiments - calculation of elastic modulus

Unconfined compression, stress-strain and stress-relaxation experiments were carried out using a commercial high precision mechanical testing system (Instron 5944, Norwood, MA, USA). The specimens were cut in an orthogonal shape with approximate dimensions 3×3×2 mm (length × width × thickness). According to the stress-strain protocol the specimens were placed between two parallel platens and they were compressed to a final strain of 30% with a strain rate of 0.05mm/min, the minimum rate the system can apply in order to avoid any transient, poroelastic effects. Stress was calculated as the force measured on the load cell divided by the initial surface area of the specimen (i.e., 1st Piola-Kirchhoff stress), and displacement data were converted to strain as $\varepsilon = \Delta l / l_0$, where Δl the change in the length of the specimen in the direction of compression and l_0 the initial, undeformed length. The elastic modulus was calculated from the slope of the stress-strain curve at 30% strain.

Stress-relaxation experiments - Calculation of hydraulic conductivity

For the stress relaxation experiments, specimens underwent four cycles of testing for each of which a 5% compressive strain was applied for 1 minute, followed by a 10 minute hold and the stress vs time response of the tissue was recorded (Supplementary Fig. S5). Subsequently a common biphasic model of soft tissue mechanics was employed [20] accounting for both the solid components (cells and extracellular matrix) and the fluid phase (interstitial fluid) of the tumor. The solid phase stress, $\boldsymbol{\sigma}^s$, was modeled as a neo-Hookean material and the values of the elastic

modulus used in the neo-Hookean model were obtained by the stress-strain experiments, assuming the Poison's ratio to be v=0.45. The fluid phase was assumed to be inviscid (i.e., ideal fluid) with the fluid stress was given by $\boldsymbol{\sigma}^f = -p\mathbf{I}$, where p the interstitial fluid pressure. The interstitial fluid velocity was described by Darcy's law, according to which the velocity is proportional to the interstitial fluid pressure gradient with a proportionality constant the hydraulic conductivity of the interstitial space, i.e.,

$$v = -K_t \nabla p \tag{15}$$

The governing equations are the momentum balance:

$$\nabla \cdot (\boldsymbol{\sigma}^s - p\mathbf{I}) = 0 \tag{16}$$

and the mass conservation

$$\nabla \cdot v^s - K_t \nabla^2 p \ = 0 \tag{17}$$

The model was implemented in the finite elements commercial software COMSOL Multiphysics version 4.4 (COMSOL, Inc., Burlington, MA). The unconfined compression, stress relaxation experiment was simulated in the mathematical model as a quasi-static problem and model predictions of the stress as a function of time were fitted to the experimental data by varying only the hydraulic conductivity of the tumor interstitial space (Supplementary Fig. S5). From the fitting the value of the hydraulic conductivity was determined. Notice that the tumor hydraulic conductivity describes the ease by which interstitial fluid percolates through the pores of the tumor interstitium and thus, it determines the transient stress response of the tumor (i.e., the peaks of the stress and the relaxation times in Supplementary Fig. S5).

Interstitial fluid pressure measurement

Interstitial fluid pressure (IFP) was measured *in vivo* with the wick-in-needle technique [17,21,22] after mice were anesthetized by intraperitoneal injection of Avertin (200mg/kg) and prior to tumor excision.

Alamar Blue assay for cell viability

4T1 cells ($2x10^5$) were seeded in 6-well plates and the following day were either mock-treated (DMSO/PBS) or treated with 50μM Tranilast or 100μM Tranilast alone or 2.5μg/ml Doxorubicin alone, or 50μM Tranilast plus 2.5μg/ml Doxorubicin or 100μM Tranilast plus 2.5μg/ml Doxorubicin for 24 hours. To calculate the percentage of viable cells at each treatment condition compared to control, 10% AlamarBlue (Invitrogen) was added in each well followed by 4 hours incubation at 37^oC/ 5% CO_2. The absorbances at 570nm and 600nm wavelengths were measured using a Microplate Reader (Ayto RT-2100C).

Statistical Analysis. The data are presented as means with standard errors (S.E.). Groups were compared using Student's t-test.

Table S1 Baseline values of model parameters

Parameter	Description	Value	Reference
k_{th}	hydraulic conductivity	$0.9\text{x}10^{-7}$ cm^2/mmHg-sec	[23]
d	vessel diameter	15 µm	[7]
d_p	Vessel wall pore size	200 nm	[25]
γ	fraction of wall surface area	1x 10^{-4}	[25]
D	drug diffusion coefficient	2.5×10^{-5} $m^2\cdot day^{-1}$ for 1nm<br>2.5×10^{-7} $m^2\cdot day^{-1}$ for 10nm<br>2.5×10^{-8} $m^2\cdot day^{-1}$ for 100nm	[24]
k_d	blood circulation decay	0.417 day^{-1}	[25]
P_v	vascular pressure	25 mmHg at the inlet<br>5 mmHg at the outlet	[26]
S_v	vascular density	200 cm^{-1}	[27]
L	vessel wall thickness	5×10^{-6} m	[28]
η	blood viscosity	3×10^{-5} mmHg·s	[28]
v	Poison’s ratio	0.45	-----

Supplementary Figures

Figure S1

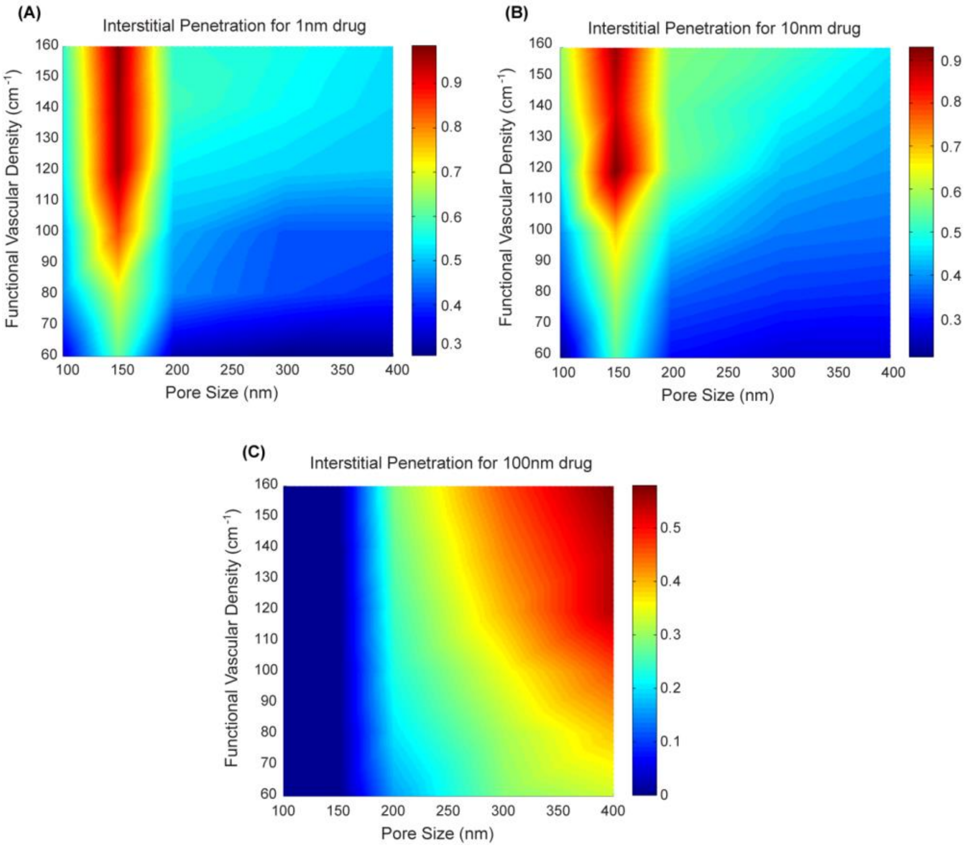

Figure S1. Model predictions for the interstitial penetration of drugs 1, 10 and 100 nm in size as a function of functional vascular density and vessel wall pore size. Interstitial penetration was quantified as the area of the interstitial space that the drug has reached in concentration higher than 5% of the concentration of the drug at the inlet of the vascular network. For the 1nm (A) and 10nm (B) drugs, decompression of the vessels with stress alleviation will increase the fraction of perfused vessels (i.e., the functional vascular density) and will improve the distribution of the drug. The benefit is optimal for pore sizes in the range of 100-200 nm. For 100nm particles (C) large pore sizes are required for the drug to cross the tumor

vessel wall from the vascular into the interstitial space and thus, the benefit of stress alleviation is optimal for large pores.

Figure S2

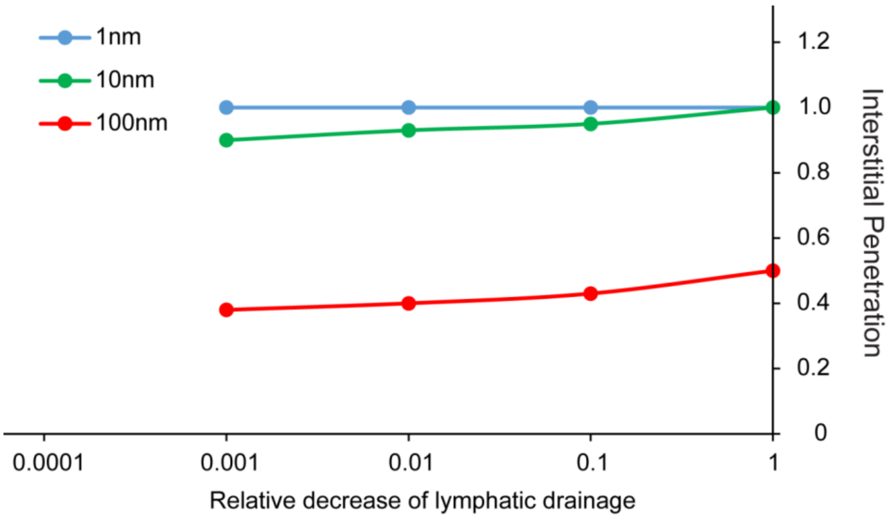

Figure S2. Interstitial penetration of drugs of 1, 10 and 100 nm in size as a function of the decrease of lymphatic drainage at the tumor periphery. Interstitial penetration was quantified as the area of the interstitial space that the drug has reached in concentration higher than 5% of the concentration of the drug at the inlet of the vascular network. Lymphatic drainage of 1 corresponds to fully functional lymphatic vessels at the tumor periphery, so that all fluid reaching the periphery to disappear (zero fluid pressure boundary condition). Subsequently, the amount of fluid that was removed from the periphery of the tumor was reduced by one, two and three orders of magnitude. There is no strong dependence of the interstitial penetration on the lymphatic function at the periphery of the tumor.

Figure S3

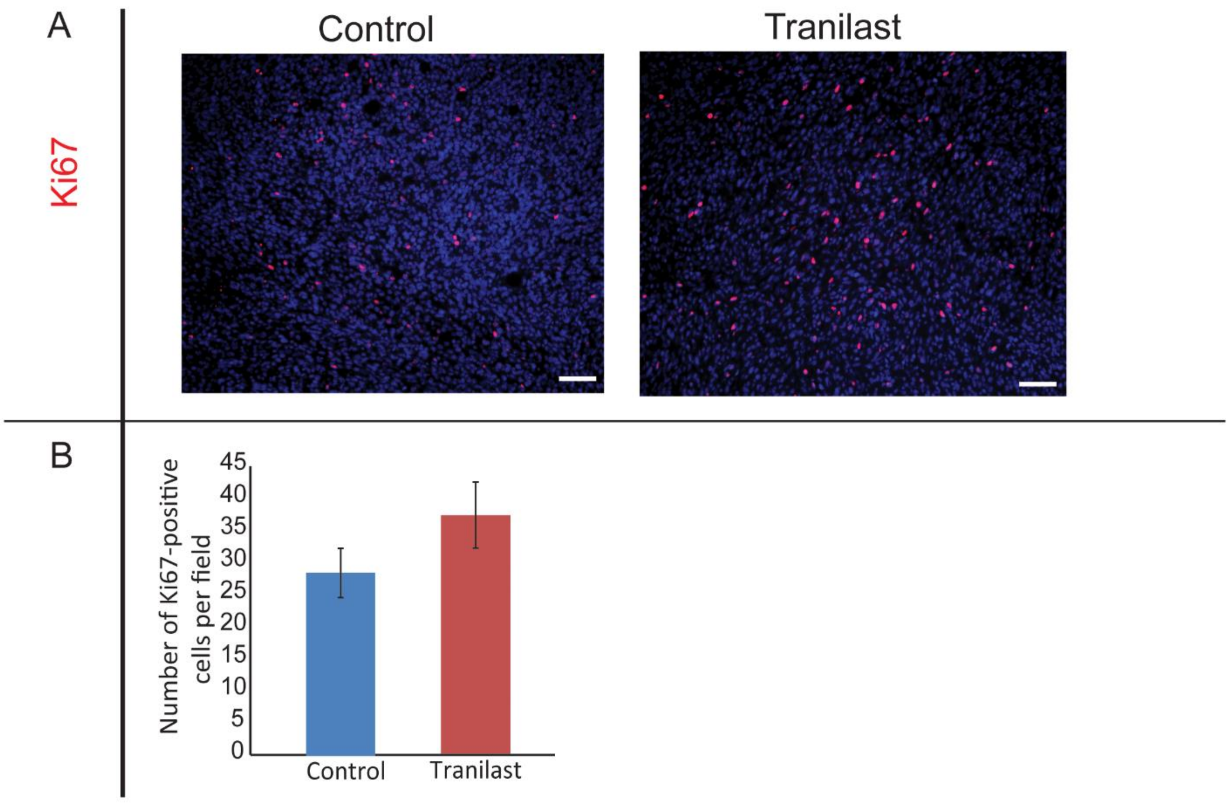

Figure S3: Tranilast does not affect cell proliferation *in vivo*. A. Representative images of paraffin-embedded tumor sections (20X), derived from control or tranilast-treated mice, stained with an antibody against Ki67 (red) and counterstained with DAPI (blue). Scale bar: 100μm. **B.** Quantification of Ki67 positive cells per field of view using a previously established house code in MATLAB (n=16) [29].

Figure S4

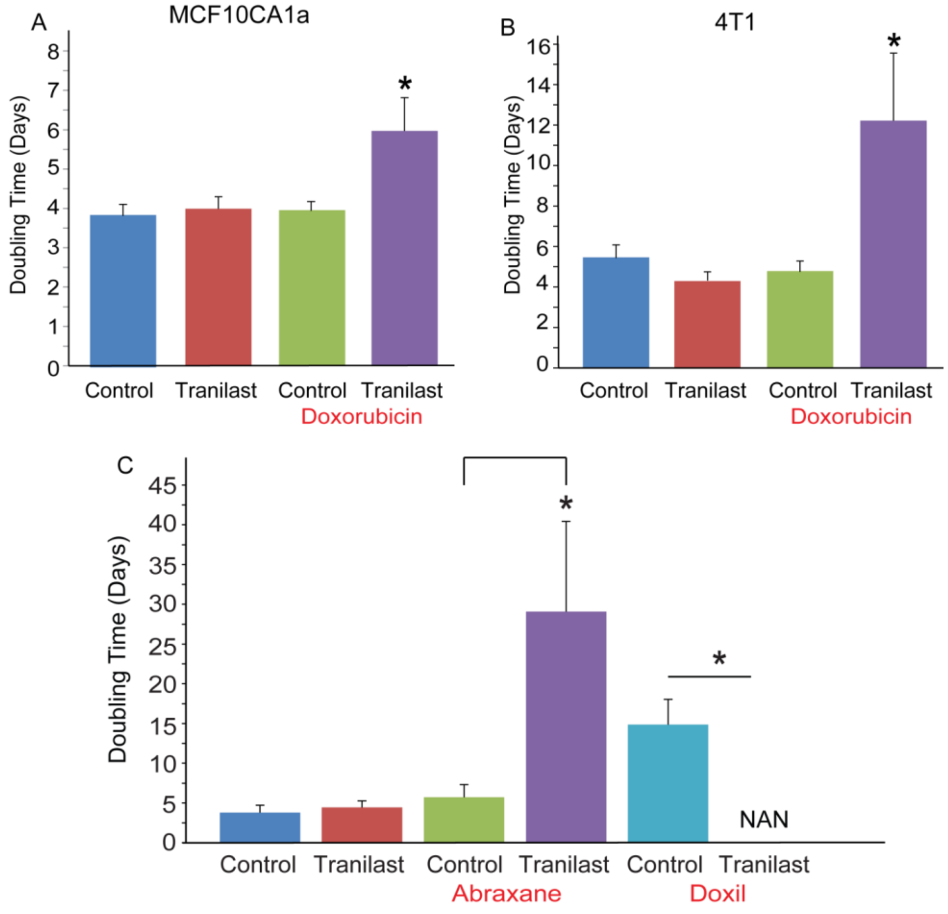

Figure S4. Combinatorial treatment of Tranilast with doxorubicin, Abraxane and Doxil significantly increases tumor volume doubling time. A-B. Tranilast enhances the effectiveness of chemotherapy, as indicated by the increase of the tumor doubling time for the MCF10CA1a and 4T1 breast tumor models compared to doxorubicin treatment alone. **C.** Tranilast increases significantly tumor doubling time of Abraxane (20mg/Kg) and Doxil (3mg/Kg) treated MCF10CA1a tumors compared to monotherapy alone. Asterisks denote a statistically significant difference. NAN stands for not available number; tumors treated with Tranilast and Doxil exhibited tumor regression and a doubling time could not be calculated.

Figure S5

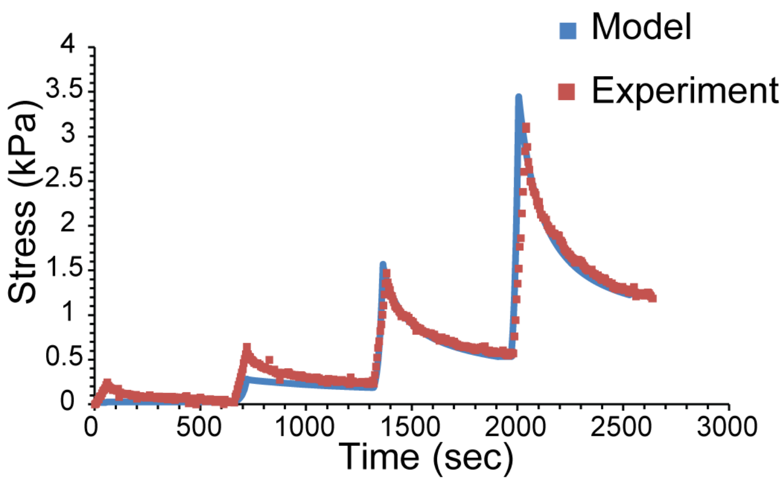

Figure S5. Typical stress vs time data of the stress relaxation experiment of the tumors tested along with the fit of the biphasic model. The only fitting parameter was the hydraulic conductivity of the interstitial space.

Figure S6

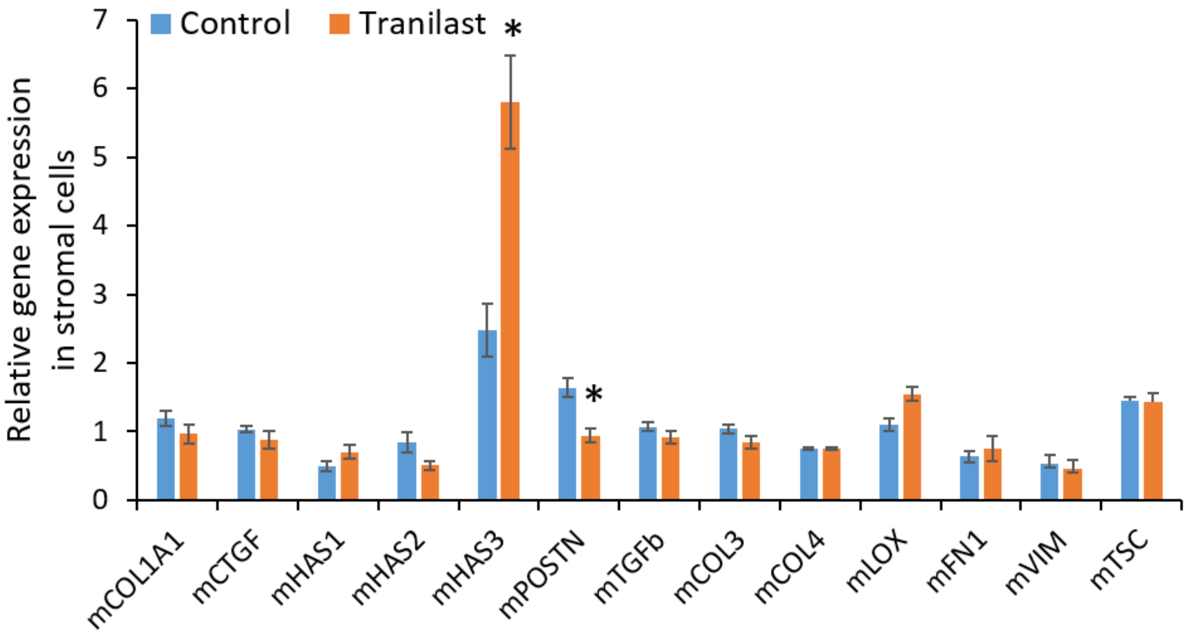

Figure S6: Effect of Tranilast on mouse stromal cells *in vivo*. Real-time PCR gene expression analysis and quantification of mCOL1A1, mCTGF, mHAS1, mHAS2, mHAS3, mPOSTN, mTGFβ, mCOL3, mCOL4, mLOX, mFN1, mVIM and mTSC mRNA levels extracted from control-treated compared to Tranilast-treated MCF10CA1a tumors, indicated that Tranilast suppresses COL1A1, CTGF, HAS2 and HAS3 gene expression. Relative expression for all genes in both groups was normalized based on the expression of beta-actin. Data represent the average of at least 3 independent experiments from 5 control and 5 tranilast-treated tumors $\pm$ S.E. values and asterisks indicate statistically significant differences between compared groups ($p < 0.05$).

Figure S7

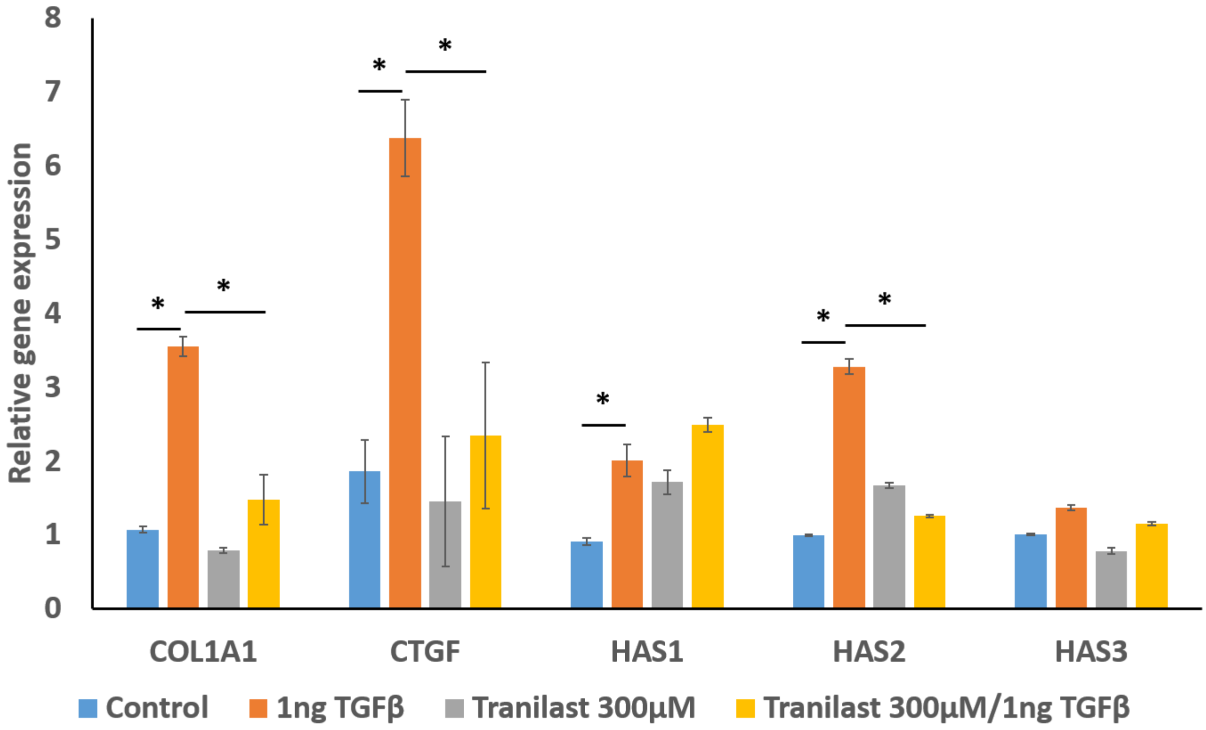

Figure S7. Tranilast inhibits TGFβ-induced expression of matrix remodeling genes in vitro. Real-time PCR gene expression analysis and quantification of COL1A1, CTGF, HAS1, HAS2 and HAS3 mRNA levels in 4T1 murine mammary carcinoma cells that were pre-treated with DMSO (control) or 300µM Tranilast followed by treatment with 1ng TGFβ or its solvent for 24 hours. Relative expression of all genes was normalized based on the expression of β-actin. Data represent the average of at least 3 independent experiments ± S.E. values and (*) indicates statistically significant differences between compared groups ($p < 0.05$).

Figure S8

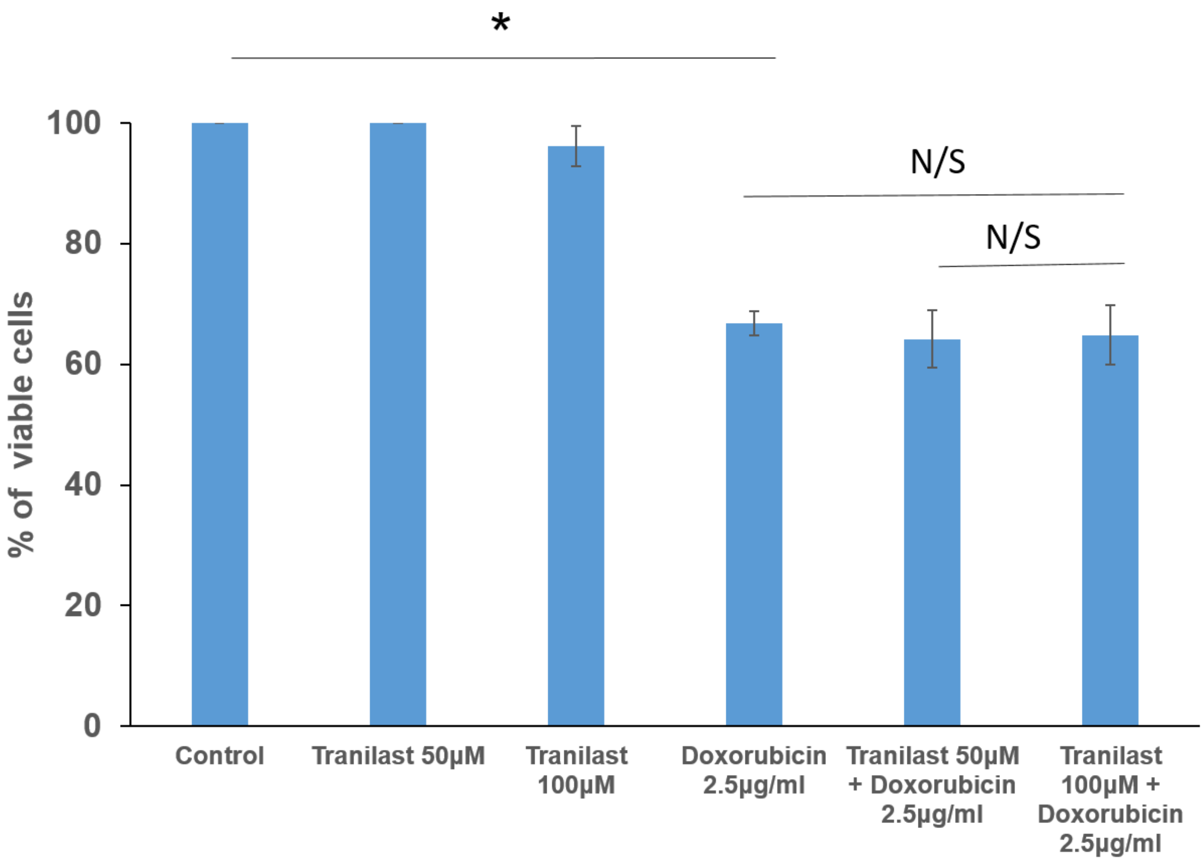

Figure S8: Tranilast does not increase sensitivity of 4T1 cells to doxorubicin *in vitro*.

Alamar blue assay for the calculation of viable 4T1 breast cancer cells in the presence of Tranilast (50µM or 100µM) alone or 2.5µg/ml Doxorubicin alone, or combination of 2.5µg/ml Doxorubicin with either 50µM or 100µM Tranilast for 24 hours. The percentage of viable cells at each treatment condition was calculated compared to mock-treated cells based on the absorbance at 570nm and 600nm wavelengths. Data represent the average of at least 3 independent experiments ± S.E. values and (*) indicates statistically significant differences between compared groups ($p < 0.05$). N/S indicates no statistical significance.

References

1 Chauhan, V. P. *et al.* Normalization of tumour blood vessels improves the delivery of nanomedicines in a size-dependent manner. *Nature Nanotechnology* **7**, 383-388 (2012).

2 Baish, J. W. & Jain, R. K. Fractals and cancer. *Cancer Res* **60**, 3683-3688 (2000).

3 Gazit, Y. *et al.* Fractal characteristics of tumor vascular architecture during tumor growth and regression. *Microcirculation* **4**, 395-402 (1997).

4 Baish, J. W. *et al.* Role of tumor vascular architecture in nutrient and drug delivery: an invasion percolation-based network model. *Microvasc Res* **51**, 327-346, doi:S0026-2862(96)90031-X [pii] 10.1006/mvre.1996.0031 (1996).

5 Baish, J. W. *et al.* Scaling rules for diffusive drug delivery in tumor and normal tissues. *Proceedings of the National Academy of Sciences of the United States of America* **108**, 1799-1803, doi:10.1073/pnas.1018154108 [pii] (2011).

6 Deen, W. M. Hindered Transport of Large molecules in Liquid-Filled Pores. *AIChE J* **33**, 1409-1425 (1987).

7 Baish, J. W., Netti, P. A. & Jain, R. K. Transmural coupling of fluid flow in microcirculatory network and interstitium in tumors. *Microvasc Res* **53**, 128-141, doi:S0026-2862(96)92005-1 [pii] 10.1006/mvre.1996.2005 (1997).

8 Hobbs, S. K. *et al.* Regulation of transport pathways in tumor vessels: role of tumor type and microenvironment. *Proc Natl Acad Sci U S A* **95**, 4607-4612 (1998).

9 Hashizume, H. *et al.* Openings between defective endothelial cells explain tumor vessel leakiness. *Am J Pathol* **156**, 1363-1380 (2000).

10 Phillips, R. J. A hydrodynamic model for hindered diffusion of proteins and micelles in hydrogels. *Biophys J* **79**, 3350-3353, doi:S0006-3495(00)76566-0 [pii] 10.1016/S0006-3495(00)76566-0 (2000).

11 Clague, D. S. & Phillips, R. J. Hindered diffusion of spherical macromolecules through dilute fibrous media. *Physics of Fluids* **8**, 1720-1731 (1996).

12 Johansson, L. & Lofroth, J. E. Diffusion and interaction in gels and solutions. 4. Hard sphere Brownian dynamics simulations. *J. Chem. Phys.* **98**, 7471-7479 (1993).

13 Amsden, B. Solute diffusion within hydrogels. Mechanisms and models. *Macromolecules* **31**, 8382-8395 (1998).

14 Netti, P. A., Berk, D. A., Swartz, M. A., Grodzinsky, A. J. & Jain, R. K. Role of extracellular matrix assembly in interstitial transport in solid tumors. *Cancer Res* **60**, 2497-2503 (2000).

15 Stylianopoulos, T., Diop-Frimpong, B., Munn, L. L. & Jain, R. K. Diffusion anisotorpy in collagen gels and tumors: The effect of fiber network orientation. *Biophys J* **99**, 3119-3128 (2010).

16 Jackson, G. W. & James, D. F. The permeability of fibrous porous-media. *Canadian Journal of Chemical Engineering* **64**, 364-374 (1986).

17 Stylianopoulos, T. *et al.* Causes, consequences, and remedies for growth-induced solid stress in murine and human tumors. *Proceedings of the National Academy of Sciences of the United States of America* **109**, 15101-15108, doi:10.1073/pnas.1213353109 (2012).

18 Laginha, K. M., Verwoert, S., Charrois, G. J. & Allen, T. M. Determination of doxorubicin levels in whole tumor and tumor nuclei in murine breast cancer tumors. *Clin Cancer Res* **11**, 6944-6949, doi:10.1158/1078-0432.CCR-05-0343 (2005).

19 Papageorgis, P. *et al.* Smad Signaling Is Required to Maintain Epigenetic Silencing during Breast Cancer Progression. *Cancer Res* **70**, 968-978 (2010).

20 Mow, V. C., Kuei, S. C., Lai, W. M. & Armstrong, C. G. Biphasic creep and stress relaxation of articular cartilage in compression? Theory and experiments. *Journal of Biomechanical Engineering* **102**, 73-84 (1980).

21 Boucher, Y., Baxter, L. T. & Jain, R. K. Interstitial pressure gradients in tissue-isolated and subcutaneous tumors: implications for therapy. *Cancer Res* **50**, 4478-4484 (1990).

22 Fadnes, H. O., Reed, R. K. & Aukland, K. Interstitial fluid pressure in rats measured with a modified wick technique. *Microvasc Res* **14**, 27-36 (1977).

23 Netti, P. A., Berk, D. A., Swartz, M. A., Grodzinsky, A. J. & Jain, R. K. Role of extracellular matrix assembly in interstitial transport in solid tumors. *Cancer Res* **60**, 2497-2503 (2000).

24 Pluen, A. *et al.* Role of tumor-host interactions in interstitial diffusion of macromolecules: cranial vs. subcutaneous tumors. *Proc Natl Acad Sci U S A* **98**, 4628-4633, doi:10.1073/pnas.081626898 081626898 [pii] (2001).

25 Chauhan, V. P. *et al.* Normalization of tumour blood vessels improves the delivery of nanomedicines in a size-dependent manner. *Nat Nanotechnol* **7**, 383-388, doi:10.1038/nnano.2012.45 [pii] (2012).

26 Boucher, Y. & Jain, R. K. Microvascular pressure is the principal driving force for interstitial hypertension in solid tumors: implications for vascular collapse. *Cancer Res* **52**, 5110-5114 (1992).

27 Yuan, F. *et al.* Vascular permeability in a human tumor xenograft: molecular size dependence and cutoff size. *Cancer Res* **55**, 3752-3756 (1995).

28 Stylianopoulos, T. *et al.* Coevolution of solid stress and interstitial fluid pressure in tumors during progression: implications for vascular collapse. *Cancer Res* **73**, 3833-3841, doi:10.1158/0008-5472.CAN-12-4521 [pii] (2013).

29 Chauhan, V. P. *et al.* Angiotensin inhibition enhances drug delivery and potentiates chemotherapy by decompressing tumour blood vessels. *Nat Commun* **4**, 2516, doi:10.1038/ncomms3516 [pii] (2013).
